# Antibiotics impact plant traits, even at small concentrations

**DOI:** 10.1093/aobpla/plx010

**Published:** 2017-03-13

**Authors:** Vanessa Minden, Andrea Deloy, Anna Martina Volkert, Sara Diana Leonhardt, Gesine Pufal

**Affiliations:** 1 Department of Biology, Ecology and Biodiversity, Vrije Universiteit Brussel, Pleinlaan 2, 1050 Brussels, Belgium; 2 Landscape Ecology Group, Institute of Biology and Environmental Sciences, Carl von Óssietzky-Strasse 9-11, 26111 Oldenburg, Germany; 3 Department of Animal Ecology and Tropical Biology, Biozentrum, Am Hubland, University of Würzburg, 97074 Würzburg, Germany; 4 Nature Conservation and Landscape Ecology, Faculty of Environment and Natural Resources, University of Freiburg, Tennenbacher Strasse 4, 79106 Freiburg, Germany

**Keywords:** *Brassica napus*, *Capsella bursa-pastoris*, germination, hormesis, penicillin, plant functional traits, sulfadiazine, tetracycline

## Abstract

Antibiotics of veterinary origin are released to agricultural fields via grazing animals or manure. Possible effects on human health through the consumption of antibiotic exposed crop plants have been intensively investigated. However, information is still lacking on the effects of antibiotics on plants themselves, particularly on non-crop species, although evidence suggests adverse effects of antibiotics on growth and performance of plants. This study evaluated the effects of three major antibiotics, penicillin, sulfadiazine and tetracycline, on the germination rates and post-germinative traits of four plant species during ontogenesis and at the time of full development. Antibiotic concentrations were chosen as to reflect *in vivo* situations, i.e. concentrations similar to those detected in soils. Plant species included two herb species and two grass species, and represent two crop-species and two non-crop species commonly found in field margins, respectively. Germination tests were performed in climate chambers and effects on the remaining plant traits were determined in greenhouse experiments. Results show that antibiotics, even in small concentrations, significantly affect plant traits. These effects include delayed germination and post-germinative development. Effects were species and functional group dependent, with herbs being more sensitive to antibiotics then grasses. Responses were either negative or positive, depending on plant species and antibiotic. Effects were generally stronger for penicillin and sulfadiazine than for tetracycline. Our study shows that cropland species respond to the use of different antibiotics in livestock industry, for example, with delayed germination and lower biomass allocation, indicating possible effects on yield in farmland fertilized with manure containing antibiotics. Also, antibiotics can alter the composition of plant species in natural field margins, due to different species-specific responses, with unknown consequences for higher trophic levels.

## Teaser

Antibiotics used in livestock industry are released to agricultural fields via grazing animals and manure. From there, they may affect human health due to consumption of antibiotic-exposed crop plants or drinking water. Also they may affect performance of natural occurring non-target species. Our study shows that antibiotics, even in small concentrations, significantly affect plant traits. Cropland species showed delayed germination and lower biomass allocation, indicating possible yield-effects in farmland fertilized with manure containing antibiotics. Antibiotics may also alter the composition of plant species in natural field margins, due to different species-specific responses, with unknown consequences for higher trophic levels.

## Introduction

Antibiotics are used to treat infections in humans and animals by either directly killing bacteria or inhibiting their growth (World Health Organization 2015; Chopra and Roberts 2001; Miller 2002). The use of antibiotics has become integral to livestock industry, with 8481 t of veterinary antibiotics sold alone in the EU/EEA (European Economic Area) in 2011 (European Medicines Agency 2013). Antibiotics applied to animals are poorly absorbed in the gut and as much as 90 % of some antibiotics may be excreted (Kumar *et al.* 2005; Winckler and Grafe 2001; Jjemba 2002). These antibiotics may be released to the environment by grazing animals or manure (Thiele-Bruhn 2003; Martinez-Carballo *et al.* 2007). Some antibiotics are highly stable in manure and soil, with residues still detectable one year after application (Thiele-Bruhn 2003). Some antibiotics may even persist for several years (Förster *et al.* 2009). For example, in agricultural landscapes with conventional land use and manure fertilization, tetracycline and sulfadiazine were detected at average soil concentrations of 10–15 µg kg^−1^ and 32–198 µg kg^−1^, respectively (Hamscher *et al.* 2000; 2002, 2005; Christian *et al.* 2003; Aust *et al.* 2008). From the farmlands, antibiotics may then be transported further to ditches, streams and rivers via runoff (Kay *et al.* 2005; Burkhardt *et al.* 2005; Stoob *et al.* 2007), to groundwater via leaching (Blackwell *et al.* 2007) or may directly be ingested by organisms (Boxall *et al.* 2006). How organisms respond to natural concentrations of antibiotics as found in soil, water and other organisms is, however, poorly understood. The majority of studies conducted for elucidating the effect of antibiotics on plants used much higher concentrations, which do not resemble *in vivo* situations (e.g. Liu *et al.* 2009 (100–500 000 µg L^−1^), Michelini *et al.* 2013 (11 500 µg L^−1^), Migliore *et al.* 2010 (5–50 000 µg L^−1^), Michelini *et al.* 2012 (10 000–200 000 µg kg^−1^)).

Whereas possible detrimental effects of antibiotics taken up by crop plants on human health have been intensively investigated (Grote *et al.* 2007; Kumar *et al.* 2005; Pan *et al.* 2014; Kang *et al.* 2013) the effect of antibiotics on plants themselves, particularly on non-crop species, has received much less attention. There is significant evidence that antibiotics adversely affect the growth and performance of plants (Migliore *et al.* 2010; Liu *et al.* 2013); however, they can also promote allometric responses (see examples in Table 1).

**Table 1:** Examples of how antibiotics affect crop plants and non-crop plants

Crop plant species			
Antibiotic	Target species	Concentration	Effect on plants/reference
Amoxicillin	Carrot (*Daucus carota*)	1–10 000 µg L^−1^	No effect on germination, despite the highest
Chlortetracycline	Lettuce (*Lactuca sativa*)		concentration; decrease of root and shoot
Levofloxacin	Alfalfa (*Medicago sativa*)		lengths at several concentrations^1^
Lincomycin			
Oxytetracycline			
Sulfamethazine			
Sulfamethoxazole			
Tetracycline			
Trimethoprim			
Tylosin			
Chlortetracycline	Corn (*Zea mays*)	0.02 µg mL^−1^	Bioaccumulation^2^
	Green Onion (*Allium cepa*)		
	Cabbage (*Brassica oleracea*)		
Chlortetracycline	Sweet Oat (*Avena sativa*)	0–500 mg L^−1^	Germination partly inhibited, decrease growth towards sulfonamides, inhibition of
Tetracycline	Rice (*Oryza sativa*)		phosphatase activity^3^
Tylosin	Cucumber (*Cucumis sativus*)		
Sulfamethoxazole			
Sulfamethazine			
Trimethoprim			
Gentamicin	Carrot (*Daucus carota*)	0, 0.5, 1 mg kg^−1^	Bioaccumulation, partly reduced growth^4^
Streptomycin	Lettuce (*Lactuca sativa*)		
	Radish (*Rhaphanus sativus*)		
Sulfadimethoxine	Millet (*Panicum miliaceum*)	300 mg L^−1^	Reduction in root and stem growth, lower number of leaves, lower biomass production^5^
	Pea (*Pisum sativum*)		
	Corn (*Zea mays*)		
Sulfamethoxine	Barley (*Hordeum vulgare*)	11.5 µg mL^−1^	Stimulation of root hair and lateral roots, increased electrolyte release from roots^6^
Sulfamethazine			
Sulfamethazine	Yellow lupin (*Lupinus luteus*)	0.01, 0.1, 0.25,	Appearance of necroses and root decay,
	Pea (*Pisum sativum*)	1, 5, 15, 20 mM	decreased activity of mitochondrial cytochrome c oxidase^7^
	Lentil (*Lens culinaris)*		
	Soybean (*Glycine max*)		
	Adzuki bean (*Vigna angularis*)		
	Alfalfa (*Medicago sativa*)		
Sulfonamide	Corn (*Zea mays*)	10, 200 µg g^−1^	Bioaccumulation, reduced stem length development, death^8^
Tetracycline	Wheat (*Triticum aestivum*)	0–100 mg L^−1^	Reduced growth of roots and stems, no effect on germination^9^
Tetracycline	Pea (*Pisum sativum*)	0–8 mg kg^−1^	Bioaccumulation, decreased peroxidase activity
Oxytetracycline			(at concentrations above 0.4 mg/kg),
Chlortetracycline			decreased root length^10^
Tetracycline	Carrot (*Daucus carota*)	0–300 mg L^−1^	Decrease in germination rates, inhibition of root
Sulfamethazine	Cucumber (*Cucumis sativus*)		and shoot elongation^11^
Norfloxacin	Lettuce (*Lactuca sativa*)		
Erythromycin	Tomato (*Lycopersicon esculentum*)		
Chloramphenicol			
Oxytetracycline	Wheat (*Triticum aestivum*)	0–0.08 mmol L^−1^	Decrease in biomass and shoot length, decreases in photosynthetic rate, transpiration rate and stomatal conductance, increase in intercellular CO_2_ concentrations^12^
**Non-crop plant species**			
Ciprofloxacin	Common reed (*Phragmites*	0.1–1000 µg L^−1^	bioaccumulation, toxic effect on root activity
Oxytetracycline	*australis*)		and leaf chlorophyll, hermetic responses at low
Sulfamethazine			concentrations (0.1–1 µg/L)^13^
Sulfonamide	Crack Willow (*Salix fragilis*)	10, 200 µg g^−1^	Bioaccumulation, reduced total chlorophyll content, reduced C/N content^14^
Sulfadimethoxine	Common amaranth (*Amaranthus retroflexus*)	300 mg L^−1^	Decrease of root length, epicotyl length, cotyledon length and number of leaves^15^
	Broadleaf Plantain (*Plantago major*)	300 mg L^−1^	
	Red Sorrel (*Rumex acetosella*)		
Sulfadimethoxine	Purple Loosetrife (*Lythrum salicaria*)	0.005–50 mg L^−1^	Toxic effect on roots, coytledons and cotyledon petioles, dose-depending response of internodes and leaf length (hormetic response)^16^
Tetracycline	Poinsettia (*Euphorbia pulcherrima*)	100–1000 ppm	Suppression of the free-branching pattern^17^

References:

^1^Hillis et al. (2011),

^2^Kumar et al. (2005),

^3^Liu et al. (2009),

^4^Bassil et al. (2013),

^5^Migliore et al. (1995),

^6^Michelini et al. (2013),

^7^Piotrowicz-Cieslak et al. (2010),

^8^Michelini et al. (2012),

^9^Yang et al. (2010),

^9^Kasai et al. (2004),

^10^Ziolkowska et al. (2015),

^11^Pan and Chu (2016),

^12^Li et al. (2011),

^13^Liu et al. (2013),

^14^Michelini et al. (2012),

^15^Migliore et al. (1997),

^16^Migliore et al. (2010),

^17^Bradel et al. (2000).

Further, responses can be dose-dependent, e.g. increased growth at lower concentrations and toxic effects at higher ones (so-called hormetic responses, see Migliore *et al.* 2010). Roots are typically most affected by and accumulate most antibiotics (Migliore *et al.* 2010), where they negatively impact on root length, root elongation and number of lateral roots with consequences for plant water uptake (Piotrowicz-Cieslak *et al.* 2010; Michelini *et al.* 2012). Further studies showed that antibiotics can alter biomass production, number of leaves, branching patterns, shoot length, internode length, root/shoot ratio, fresh/dry weight, C/N and K:Ca ratio etc. (Bradel *et al.* 2000; Liu *et al.* 2009; Yang *et al.* 2010; Michelini *et al.* 2012; Li *et al.* 2011). Physiological traits affected by antibiotics are for instance photosynthetic rate, chloroplast synthase activity, transpiration rate, stomatal conductance and synthesis of abscisic acid (ABA) (Kasai *et al.* 2004; Werner *et al.* 2007). These studies clearly demonstrate that various antibiotics in the soil can be accumulated in plant tissues and have either detrimental or enhancing effects on functional traits of crop and wild plant species. They also show that effects depend on plant species, plant organ, type of antibiotic applied and its concentration. However, these studies were conducted under artificial conditions with mostly unnaturally high antibiotic concentrations, not necessarily mirroring *in vivo* conditions. Whether these effects also occur for lower antibiotic concentrations remains largely unclear.

To address this knowledge gap, we studied the effect of three antibiotics with different action modes (i.e. penicillin, tetracycline and sulfadiazine) on four plant species, including crop (*Brassica napus* and *Triticum aestivum*) and non-crop (*Capsella bursa-pastoris* and *Apera spica-venti*) species. Both crop species (*B. napus* and *T. aestivum*) belong to the most commonly grown crops worldwide (Leff *et al.* 2004; FAO—Food and Agriculture Organization of the United Nations 2016) and are highly likely exposed to antibiotics due to fertilization of crop fields with slurry or manure. The non-crop species (*C. bursa-pastoris* and *A. spica-venti*) are commonly found along most crop field margins in Germany and are likely unintentionally exposed to antibiotic charged manure applied to fields (Ellenberg and Leuschner 2010). We applied concentrations of antibiotics as previously reported for grasslands (from now on referred to as natural concentrations, Thiele-Bruhn 2003) to plants grown in greenhouses and measured germination rates and plant functional traits during ontogenesis and at fully developed plant individuals.

Specifically, we asked (i) whether natural concentrations of antibiotics affect both germination and functional traits of plants, and (ii) whether trait responses were more similar among crop and non-crop plant species than between crop and non-crop species (i.e. *B. napus* and *T. aestivum* versus *C. bursa-pastoris* and *A. spica-venti*) or among herbs and grasses (e.g. *B. napus* and *C. bursa-pastoris*) than between herbs and grasses.

Given the low concentration rates and the three antibiotics differing in their action modes used in this study we allowed for the following expectations: germination rates and functional trait responses (i) could be negatively affected as reported by other studies, (ii) could be unaffected and not differ from control treatments and (iii) could be higher than the control treatments. The latter would point to a hormetic response with increased values in lower treatments.

## Methods

### Selected species

Two crop species and two non-crop species were chosen, with one representative of either group belonging to the family of Brassicaceae (*B.napus* (summer rapeseed) and *C.bursa-pastoris* (shepherd’s purse)) or Poaceae (*T.aestivum* (wheat) and *A.spica-venti* (loose silky-bent)). By comparing closely related species we minimized a potential bias associated with phylogenetic distances or differences in life-history or dispersal mode (congeneric or phylogenetic approach, Burns 2004; van Kleunen *et al.* 2010). All species were annuals. Our choice further allowed comparison between crop plant/non-crop plant within the functional groups of herbs (Brassicaceae) and grasses (Poaceae), respectively.

Seeds of the plants were ordered in April 2015 from Rieger-Hofmann^®^, Sämereien Jehle (both Germany) and Botanik Sämereien, Switzerland.

### Selected antibiotics and their modes of action

The three antibiotics used in this study were penicillin G sodium salt (C_16_H_17_N_2_NaO_4_S), sulfadiazine (C_10_H_10_N_4_O_2_S) and tetracycline (C_22_H_24_N_2_O_8_). These compounds are the most commonly sold antibiotic compound classes for food-producing species in Europe with 37 %, 23 % and 11 % of sold antibiotics, respectively (European Medicines Agency 2013). They are all polar (with logKW < 3) and thus likely accumulate in plant tissue (Trapp and Eggen 2013). Using polar antibiotics and concentrations resembling those measured in grasslands (Thiele-Bruhn 2003) should therefore ensure responses of plants to treatments and applicability of research results to *in vivo* situations. The selected antibiotics further differ in their action modes with expected different effects on plants traits, enabling us to relate specific results to a specific type of antibiotic. Penicillin G belongs to the group of β-lactam antibiotics which inhibit the biosynthesis of peptidoglycan during cell division and thus inhibits cell wall synthesis (Miller 2002; Hammes 1976). Sulfadiazine inhibits the growth of bacteria without their destruction (bacteriostasis) (Henry 1944). Tetracycline is an anti-infective agent inhibiting protein synthesis by preventing the attachment of aminoacyl-t-RNA to the ribosomal acceptor (Chopra and Roberts 2001). For known examples for effects of these antibiotics on plants see Table 1.

Biodegradation differs between different types of antibiotics. The three antibiotics in this study have been shown to remain stable in soil samples across time periods that extend the period of this experiment (i.e. 8 weeks, see Kumar *et al.* 2005; Hamscher *et al.* 2002, 2005; Christian *et al.* 2003).

### Experimental design

Plants were treated with 1 µg, 5 µg and 10 µg antibiotic/L for penicillin (P1, P5 and P10), sulfadiazine (S1, S5 and S10) and tetracycline (T1, T5 and T10), as well as with two nitrogen addition treatments (N5 and N10, see below) and one control treatment (distilled water, C). To avoid confounding effects of mixtures of antibiotics, these compounds were added as separate treatments. Converted to the amount of sand in the pots, treatments correspond to 0.038 µg kg^−1^, 0.19 µg kg^−1^ and 0.38 µg kg^−1^ sand (see description of greenhouse experiment below).

Antibiotics were ordered at Alfa Aesar (Karlsruhe, Germany). Antibiotic solutions were prepared by dissolving 1 mg of antibiotic in 1 L distilled water, and further filling up 1 mL (5 mL and 10 mL) of removed solution to 1 L volume with distilled water; pHs of all solutions were 5.5.

Each antibiotic used contains a nitrogen group. One molecule penicillin contains 7.8 % N, tetracycline contains 6.3 % N and sulfadiazine 22.4 % N. To differentiate between potential plant responses to antibiotics and/or to nitrogen provided by antibiotic degradation, we included two nitrogen (N-)treatments. Concentrations in the N-treatments were chosen to represent the amounts of nitrogen provided by the specific antibiotics in the 5 µg L^−1^ treatment (N5, pooled for penicillin and tetracycline) and in the 10 µg L^−1^ treatment (N10, for sulfadiazine). For the nitrogen treatment N5, 2.15 mg NaNO_3_ were diluted in 1 L distilled water and 1 mL of this solution was further diluted with 1 L distilled water. The same was done for the N10 treatment using 13.58 mg NaNO_3_.

Macro- and micronutrients (N, P, K, Ca, Mg, Fe, Cu, S, B, Mn, Zn, Mo) were equally applied to each experimental pot (5 mL solution/week). Nitrogen was applied as NaNO_3_, phosphorus as NaH_2_PO_4_. Composition of nutrient solutions followed Güsewell (2005), pH was adjusted to six.

#### Germination experiment

For each plant species a total of 100 seeds per treatment were germinated with simultaneous application of antibiotics, nitrogen solution (N5 and N10) and distilled water (C), respectively **[see ****Supporting Information—Fig. S1****]**.

Seeds were stratified following Anandarajah *et al.* (1991) for *B. napus* (4 °C for 10 days) and Toorop *et al.* (2012) for *C. bursa-pastoris* (4 °C for 3 days). For *T.aestivum* and *A.spica-venti* no specific treatment is reported in the literature, except soaking of seeds prior to sowing for *T. aestivum* (Siddiqui *et al.* 2009) and storing under dry conditions for *A. spica-venti* (Wallgren and Avholm 1978).

We placed 25 seeds on filter paper in 90 mm × 90 mm petri dishes, with four replicates per plant species and treatment, resulting in 192 trials. Filter papers were treated with 5 mL of the respective treatment solution. Petri dishes were covered and kept in a dark climate chamber set to 24 °C. Germination success was evaluated using the length of the radicle (> 2mm). Germination success was controlled each day for 14 days in total and the corresponding seed was sorted out of the petri dish and discarded from the remaining experiment.

#### Greenhouse experiment

Ten individuals per plant species were exposed to a given treatment, summing up to 120 individuals per species and 480 individuals in total **[see ****Supporting Information—Fig. S1****]**. Plants were raised from seeds in germination pots with germination soil (Gartenkrone, Germany), individual plants were planted in 400 mL pots filled with quartz sand (Vitakraft, Germany) starting of June 2015 about three weeks after sowing (*B.napus* was planted in 2-L pots). To guarantee a homogenous substrate for all treatments and thus to prevent variation in soil-related factors (e.g. water-holding capacity) across pots to affect our results, we used quartz sand instead of potting soil. We mixed 25 mL of antibiotic and/or nitrogen solution with the sand before the seedlings were planted (125 mL for the 2-L pots). The volume was equivalent to the quantity held back by the quartz sand without draining. To avoid leaching of the antibiotics from pots, distilled water was filled only into saucers. Nutrient solutions were provided once a week for eight weeks. Control treatments received only distilled water and nutrients. Additionally, initial biomass was determined for each species by collecting ∼30 seedlings per species, separating leaves, stems and roots, drying them at 70 °C for 72 h and finally weighing dried samples.

At the end of the experiment (i.e. after eight weeks), plant individuals were harvested and separated into leaves, stems and roots, which were dried at 70 °C for 72 h and weighed. Relative growth rates (RGR) of aboveground, belowground and total biomass were calculated as RGR = (log*W*_2_ − log*W*_1_)/(*t*_2_ − *t*_1_), with *W*_2_ and *W*_1_ representing the biomass at the sequential times *t*_2_ and *t*_1_, respectively (in days, Hunt 1990, see Table 2 for overview of measured traits). Canopy height was measured biweekly (i.e. four times in the total course of the experiment) as the distance between the pot surface and the highest fully developed leaf of each plant individual (Pérez-Harguindeguy *et al.* 2013). Stem length was assessed as the total length of the aboveground shoot at the time of harvest (in cm, for *B. napus* and *C. bursa-pastoris*, not applicable for the two grass species).

**Table 2:** Measured plant traits, abbreviations and units

Plant trait	Abbreviation	Unit	Trait representative of
Relative growth rate of aboveground biomass	RGR_AGB_	mg mg^-1^ day^−1^	Growth rate
Relative growth rate of belowground biomass	RGR_BGB_	mg mg^−1^ day^−1^	Patterns
Relative growth rate of total biomass	RGR_Total_	mg mg^−1^ day^−1^	
Dry weight of leaves (live and dead)	Leaf	mg	Biomass allocation
Dry weight of stems	Stem	mg	
Dry weight of roots	Root	mg	
Canopy height	CH	cm	Growth rate and
Stem length	StemL	cm	competition related
Chlorophyll content	Chl	µg mg^−1^	plant traits
Specific Leaf Area	SLA	mm^2^ mg^−1^	
Number of live leaves	Leaf_live_	number	Turnover rates
Number of dead leaves	Leaf_dead_	number	
Root:Shoot ratio	R:S ratio		
Specific Root Length	SRL	mm mg^−1^	Traits related to
Total Root Length	TRL	mm	Nutrient uptake
Secondary Roots	SecR	n cm^−1^	
Length of Primary Root	LPR	cm	

Chlorophyll content was also measured biweekly and was determined with a Chlorophyll Meter 502-SPAD Plus (Konica Minolta, Munich, Germany), which calculates an index in ‘SPAD units’ based on absorbance at 650 and 940 nm, with an accuracy of ±1.0 SPAD units (Richardson *et al.* 2002). At each measurement date, three SPAD measurements were taken from one leaf of each individual. To obtain total chlorophyll content, as determined at the time of harvest (Lichtenthaler 1987; Lichtenthaler and Wellburn 1983) additional plant individuals of every species and treatment were raised in extra pots to provide leaf material for wet chemical analysis. Leaf samples were collected and the area of 250 mg fresh material determined (flatbed scanner and computer software ImageJ, Rasband 2014). Plant material was grinded in a mortar together with 10 mL acetone (80 %) and sea sand (VWR Chemicals) and subsequently filtered through a glass frit. The filtrate was then filled up to 20 mL by adding acetone. Absorbance of the solutions was measured with a UV/Visible spectrophotometer (Genesys 10 UV, Thermo Spectronic, Braunschweig, Germany) at 656 and 663 nm. Total chlorophyll (Total chl, µg/mg) concentrations were referred to leaf dry weight by converting dry weights of scanned leaves to leaf area via regression. Slopes and intercepts for chlorophyll content (µg/mg dry weight) versus SPAD units were calculated via ordinary least square regression and used to convert SPAD units for all individuals of the experiment into chlorophyll content.

Specific Leaf Area (SLA) was calculated as the mean area of two leaves divided by their mean dry weight (mm^2^ mm^−1^, Pérez-Harguindeguy *et al.* 2013). Two leaves per individual were collected to measure dry weight and area (flatbed scanner and computer software ImageJ, Rasband 2014). Living and dead leaves were separated and their number determined for each plant individual. If dead leaves occurred during the experiment, they were collected and added to the number of dead leaves at the end of the experiment. We also assessed the biomass allocated to belowground and aboveground plant parts (Root:Shoot), respectively, which reflects either stronger allocation towards belowground organs (values >1) or towards aboveground organs (values <1).

To measure Specific Root Length (SRL), i.e. the ratio of root length to dry mass of fine roots (<2 mm diameter), a 10 cm section of root was separated from the remaining roots, dried (70 °C, 72 h) and its weight determined (Pérez-Harguindeguy *et al.* 2013). Total Root Length was calculated from SRL and belowground biomass. Secondary Roots were counted along the 10 cm root section and number of Secondary Roots per 1 cm determined. Length of Primary Root was measured for *B. napus* and *C. bursa-pastoris* only, as primary roots in grass species degenerate in the course of ontogenesis.

Canopy height and chlorophyll content were measured every two weeks, four times in total. The remaining 15 traits were determined after the final harvest.

### Statistical analysis

All statistical analyses were done with the computer software R (R Core Team 2014). Packages used were survival (survfit(), Therneau and Grambsch 2000), geoR (Ribeiro and Diggle 2015), car (Fox and Weisberg 2011), nortest (Gross and Ligges 2015) and ggplot2 (Wickham 2009).

#### Germination experiment

To test for differences in germination rates between control and treatments, Kaplan–Meier Survival analysis was performed, which estimates the survival function for exact time events. The Kaplan–Meier estimator *Ŝ*_(__*t*__)_ was used to calculate non-parametric estimates of the survivor function

$${Ŝ}_{\left(t\right)}\;=\prod_{j=1}^{s}\left(1-\frac{d_{j}}{n_{j}}\right)$$

with *d_j_* being the number of individuals that experienced the event (i.e. here germination) in a given interval and *n_j_* the number at risk (i.e. all individuals). Differences between groups (control versus treatment) were calculated using the log-rank test (Kaplan and Meier 1958; McNair *et al.* 2012; Kleinbaum and Klein 2012).

#### Greenhouse experiment

To test for effects of antibiotics and concentration (and their interactions) on response variables (i.e. plant traits), analysis of variance (ANOVA) was carried out with species, antibiotics and concentration as factors with four and three levels, respectively. We always tested residuals for normal distribution and variances for homogeneity for each trait and for each species, and transformed the data where applicable (log-, square root- or boxcox-transformation).

Because differences in traits were strongly species specific and the antibiotic were applied independently from each other, we also tested the treatment effects separately for each species and antibiotic. We also tested for significant differences between the nitrogen treatments and the control, with the hypothesis that nitrogen addition in such small amounts should not have an effect on plant traits. As there were no significant differences, the data of the nitrogen treatments and the control treatment were pooled into one control treatment in subsequent analyses. For the two traits which were measured repeatedly during the experiment (i.e. canopy height and chlorophyll content) we performed paired *T*-tests for dependent data and tested whether antibiotics had a significant effect on the respective trait at each date of measurement.

## Results

### Germination experiment

Within the 14 days of the germination experiment, *B.napus* and *T. aestivum* germinated most rapidly, irrespective of treatment, with a mean of 1.9 days (i.e. 45 h) and 1.5 days (36 h) across all treatments, respectively. *C.bursa-pastoris* germinated latest and very poorly (Table 3), with a mean of 14.2 days and no effects of any treatment. Absolute rates of germination were highest in *T. aestivum* (99–100 %), followed by *B. napus* (93–100 %) and *A. spica-venti* (81–94 %). When germination was compared within plant species for different treatments, we found germination to be generally delayed in three of our four plant species when seeds were exposed to higher concentrations of antibiotics (except for T1 in *B. napus* which germinated earlier than the control, see Table 3). For *T. aestivum* and *A. spica-venti*, all treatments but the lowest ones (P1, S1 and T1) resulted in a significant delay of germination, with the most severe delay of 1.9 days (i.e. 45 h) at T10 in *A. spica-venti*. Interestingly, the nitrogen treatments also produced a delay in germination in *T. aestivum* and *A. spica-venti*.

**Table 3:** Results of Kaplan–Meier survival analysis for germination rates for the four plant species. Given are the mean days until germination for each treatment (with corresponding hours in brackets) and germination rates in percent. Bold numbers indicate significant differences to control treatment (*P* < 0.05), green shading indicates earlier germination, red shading indicates delayed germination of the treatment compared with control group. Treatments were: nitrogen (N5 and N10, i.e. 5 and 10 µg L^−1^), penicillin (P1, P5 and P10, i.e. 1, 5 and 10 µg L^−1^), sulfadiazine (S1, S5 and S10, i.e. 1, 5 and 10 µg L^−1^) and tetracycline (T1, T5 and T10, i.e. 1, 5 and 10 µg L^−1^)

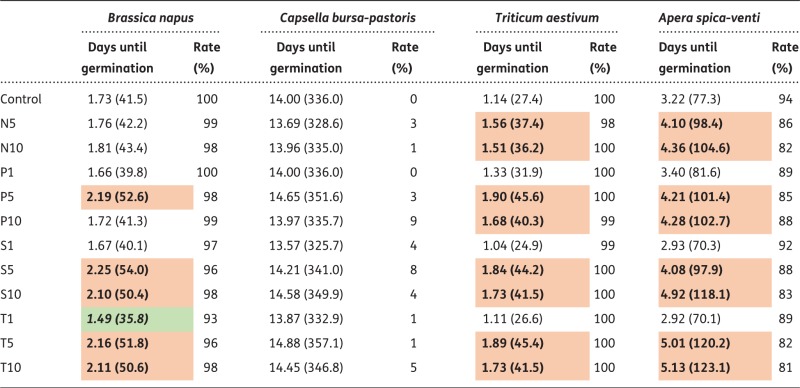

### Greenhouse experiment

Species as factor showed the strongest effect on almost all plant traits (see *F*-values in Table 4). Mean trait values were highest in *C. bursa-pastoris*, followed by *B. napus* and *A. spica-venti*, whereas eight out of twelve measured trait values were lowest in *T. aestivum* (StemL, SecR and LPR not measured for the two grass species, **[see ****Supporting Information—Table S1]**). Whereas the effect of species as factor was most pronounced, those of antibiotic and concentration were less strong (Table 4). However, every plant trait responded significantly to an interaction between either S × A (Species × Antibiotic, i.e. RGR_BGB_, Leaf, Root, R:S and SRL), S × C (Species × Concentration, i.e. RGR_AGB_, RGR_Total_ and LPR) or A × C (Antibiotic × Concentration, i.e. Stem and StemL).

**Table 4:** *F*-values, degrees of freedom and significance levels for multi-factor ANOVA analyses testing the effects of plant species, antibiotic, concentration and their interactions on different plant traits of *Brassica napus*, *Capsella bursa-pastoris*, *Triticum aestivum* and *Apera spica-venti*. Stem length (StemL), number of Secondary Roots (SecR) and Length of Primary Root (LPR) were only tested for *Brassica napus* and *Capsella bursa-pastoris*, see text. For trait description see Table 2. Significance levels: * = *P* < 0.05, ** = *P* < 0.01, *** = *P* < 0.001

Source	DF	RGR_AGB_	RGR_BGB_	RGR_Total_	Leaf	Stem	Root	SLA	Leaf_live_
Species (S)	3, 476	2194.40***	1139.89***	1759.73***	236.62***	398.43***	203.17***	159.33***	296.86***
Antibiotic (A)	2, 477	0.47	0.33	0.37	0.94	0.77	0.80	0.14	1.23
Concentration (C)	2, 477	2.96	2.17	3.07*	1.38	0.72	2.16	0.72	1.68
S × A	6, 468	0.97	2.17*	1.09	2.67*	0.42	6.38***	0.71	1.46
S × C	6, 468	2.38*	1.85	2.59*	0.96	0.11	0.96	1.08	0.87
A × C	4, 471	0.92	0.96	1.06	0.64	3.70**	0.68	0.43	1.24
S × A × C	12, 444	0.53	0.55	0.68	1.26	1.31	1.06	0.88	1.83*

**Source**	**DF**	**Leaf_dead_**	**R:S**	**SRL**	**TRL**	**DF**	**StemL**	**SecR**	**LPR**

Species (S)	3, 476	67.30***	26.43***	44.55***	65.78***	1, 238	4.14*	0.86	85.42***
Antibiotic (A)	2, 477	6.78**	0.39	4.42*	1.33	2, 237	1.98	4.81**	1.44
Concentration (C)	2, 477	2.37	0.54	0.88	3.64*	2, 237	0.51	0.97	2.15
S × A	6, 468	1.96	5.23***	3.86**	1.60	2, 234	1.10	0.06	1.21
S × C	6, 468	1.66	0.53	1.83	1.90	2, 234	1.07	1.17	3.59*
A × C	4, 471	1.57	0.47	1.10	0.99	4, 231	5.51***	1.43	0.52
S × A × C	12, 444	2.27**	1.16	1.17	1.24	4, 222	2.08	1.81	2.11

To further elucidate the specific effects of each antibiotic on plant traits, we tested for significant differences on plant traits between the control treatment and each antibiotic treatment.

**Figure 1:**
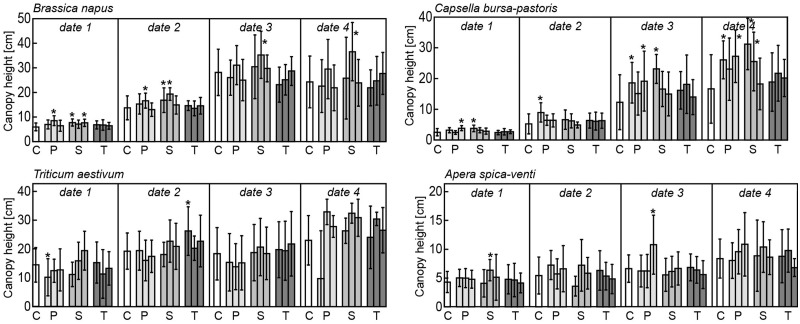
Means and standard deviations of canopy height (cm) for the four times of measurement (date 1–4) for *Brassica napus*, *Capsella bursa-pastoris*, *Triticum aestivum* and *Apera spica-venti*. Significant differences to control treatment within each date of measurement are indicated by asterisks with *P* < 0.05. C: control, P: penicillin treatment in the order 1, 5 and 10 µg L^−1^, S: sulfadiazine treatment in the order 1, 5 and 10 µg L^−1^, T: tetracycline treatment in the order 1, 5 and 10 µg L^−1^.

Canopy height of all four plant species increased in the course of the experiment. Whereas the two grass species hardly responded to any antibiotic applied (i.e. no significant differences in the trait means between the control treatment and the antibiotic treatment), the canopy height of the two herb species differed significantly from the control plants (Fig. 1). Responses were significant for penicillin and sulfadiazine, but not for tetracycline. With regard to penicillin, *B. napus* responded only at the earliest two stages of measurement and only to treatment P5, whereas *C. bursa-pastoris* responded primarily at the latest two stages of measurement and to treatments P1 and P10, respectively. *B. napus* plants treated with sulfadiazine showed significant responses throughout the course of the experiment, stronger towards S1 and S10 in the earlier stages and more pronounced towards S5 in the later stages. Individuals of *C. bursa-pastoris* responded primarily to S1 at all times of measurement despite date 2.

**Figure 2:**
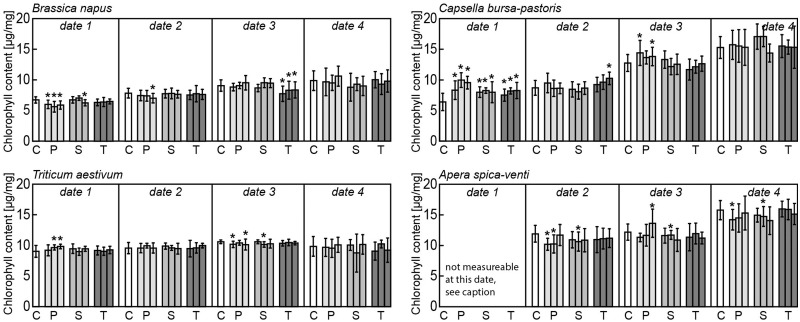
Means and standard deviations of total chlorophyll content (µg mg^−1^) for the four measurements (date 1–4) for *Brassica napus*, *Capsella bursa-pastoris*, *Triticum aestivum* and *Apera spica-venti*. Significant differences to control treatment within each date of measurement are indicated by asterisks with *P* < 0.05. C: control, P: penicillin treatment in the order 1, 5 and 10 µg L^−1^, S: sulfadiazine treatment in the order 1, 5 and 10 µg L^−1^, T: tetracycline treatment in the order 1, 5 and 10 µg L^−1^. SPAD values of *A. spica-venti* leaves could not be determined at the first date of measurement, as leaf blades were too thin for the measurement device.

Measurements of total chlorophyll content showed opposing patterns in the herb species, with *B. napus* showing decreased and *C. bursa-pastoris* increased pigment content compared with the control (Fig. 2). When treated with penicillin and tetracycline, *B. napus* had significantly lower chlorophyll content in the earlier and later stage of the experiment, respectively. In contrast, chlorophyll content of *C. bursa-pastoris* was predominantly influenced at the earliest time of measurement by all three antibiotics.

*T.* *aestivum* and *A. spica-venti* responded to penicillin and sulfadiazine, but not to tetracycline. Responses were significant both at earlier and at later stages of measurement, and pigment content was mostly lower than in the control treatment.

For the 15 plant traits determined after the final harvest, 33 % of all statistical tests performed (for all plant species) yielded significant results when plants were treated with penicillin (53 out of 162 tests), 19 % when treated with sulfadiazine (31 out of 162) and 10 % (16 out of 162) when treated with tetracycline (Tables 5 and 6; results of test statistic and means and relative standard deviations for all treatments can be found in **[****Supporting Information—Table S1****]**). For the significant results, the direction of response, i.e. whether trait means were higher or lower in the treatments than in the control, was balanced for penicillin, with 28 mean trait values being higher than in the control treatment and 25 mean trait values being lower, respectively. For sulfadiazine, mean trait values tended to be higher than in the control (21 higher, 10 lower), whereas the opposite was observed for tetracycline (three higher and 13 lower).

**Table 5:** Results of *t*-tests (*P* < 0.05) for each trait for *Brassica napus*, *Capsella bursa-pastoris*, *Triticum aestivum* and *Apera spica-venti*. Means are given for control treatment. Arrows indicate significant differences to control treatment, red arrows pointing down indicate lower values, green arrows pointing up indicate higher values within the treatment comparisons. For means and relative standard deviations of all treatments, see Table 6

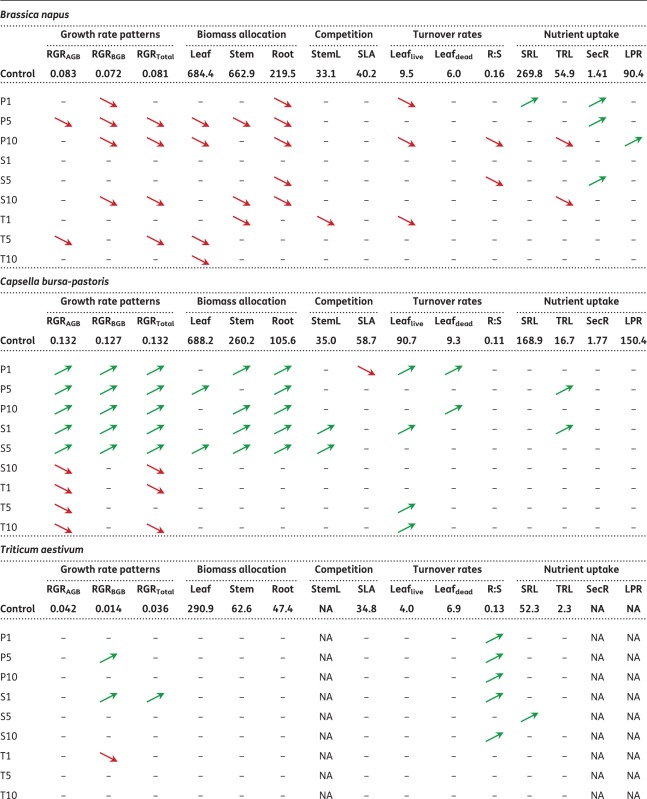
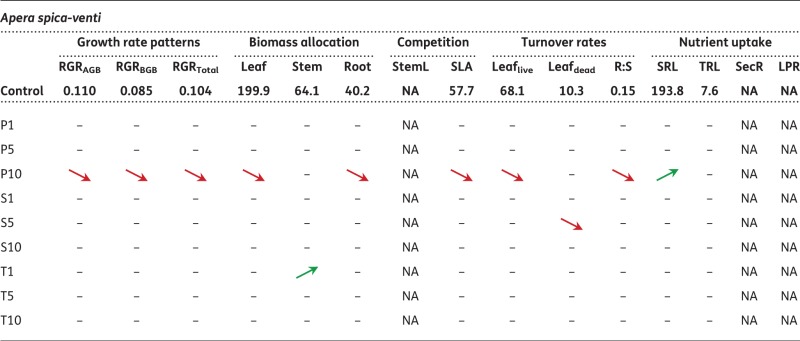

**Table 6:** Test statistics for each trait for *Brassica napus, Capsella bursa-pastoris, Triticum aestivum* and *Apera spica-venti*. Given are t-values and significance levels for the comparisons between mean trait values between control treatment and respective antibiotic treatment. Green shading indicates significantly lower values to control treatment, red shading indicates significantly higher values compared with control. ***P<0.001 **P<0.01; *P<0.05. Treatments: Control; P1, P5, P10: penicillin treatment in the order 1, 5 and 10 µg L^−1^; S1, S5, S10: sulfadiazine treatment in the order 1, 5 and 10 µg L^−1^; T1, T5, T10: tetracycline treatment in the order 1, 5 and 10 µg L^−1^. For abbreviations of traits see Table 2

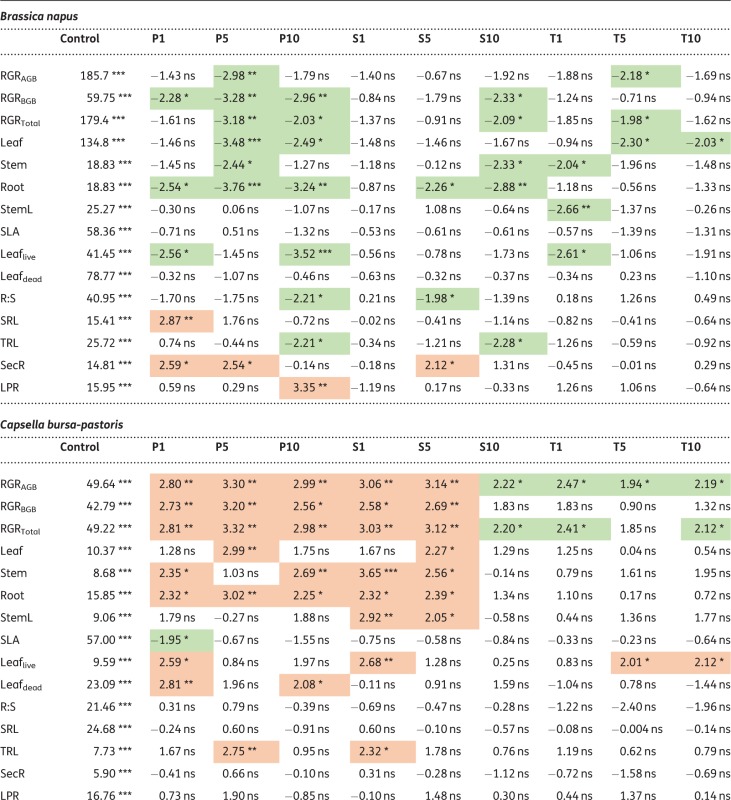
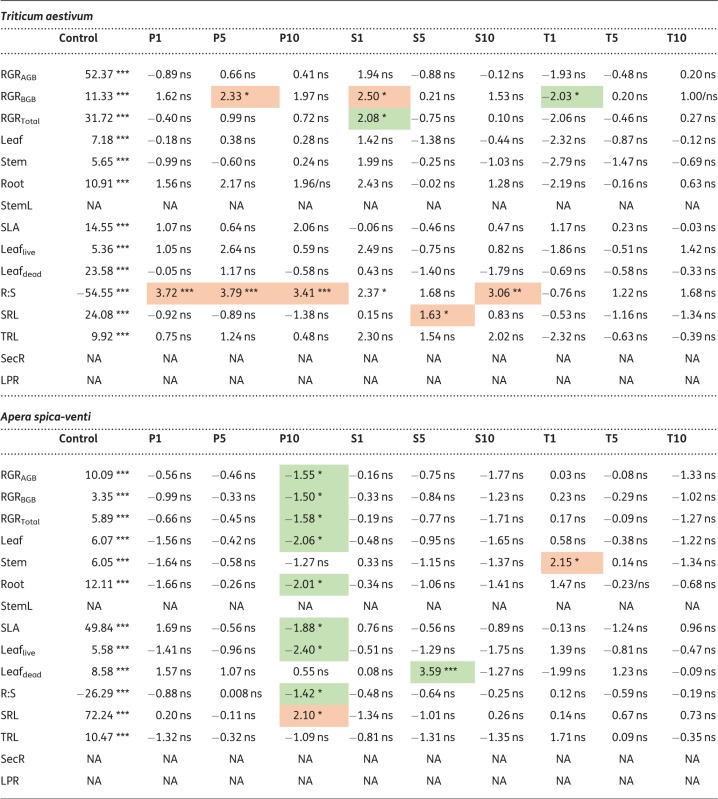

Across species, trait responses were most pronounced for penicillin. In both *B. napus* and *C. bursa-pastoris*, 44 % of all traits showed a significant response to the penicillin treatments, 9 % in *T. aestivum* and 20 % in *A. spica-venti.* The responses towards the other two antibiotics were less pronounced: 18 % of all *B. napus*-traits responded significantly to sulfadiazine (*C. bursa-pastoris* 38 %, *T. aestivum* 17 % and *A. spica-venti* 3 %) and 16 % of *B. napus*-traits to tetracycline (16 %, 3 % and 3 % in the remaining species).

The response direction differed between species. In *B. napus*, most trait values decreased under the influence of antibiotics (30 out of 35, Table 5). Traits related to growth (RGR and biomass allocation) were most affected compared with other traits and were more strongly affected by penicillin than by the other two antibiotics. The latter was also true for *C. bursa-pastoris*: growth and biomass related traits responded most strongly to the treatments, and most strongly to penicillin and sulfadiazine. However, opposite to *B. napus*, *C. bursa-pastoris* showed an increase in biomass production (except for tetracycline).

Compared with the herb species, the two grass species showed only weak responses to antibiotics (Tables 5 and 6). The most pronounced results were found for *T. aestivum* which showed a slight increase in growth and a shift towards higher biomass allocation to belowground parts (higher Root:Shoot ratio) when exposed to antibiotics. *A.spica-venti* on the other hand only responded to penicillin (with one exception each for the other two antibiotics). When treated with penicillin, eight out of 12 mean trait values were lower, and one was higher than the control, but only in the highest penicillin treatment (P10).

## Discussion

Although antibiotics in plants have been intensively studied in the context of possible detrimental effects on human health (Grote *et al.* 2007; Kumar *et al.* 2005; Pan *et al.* 2014; Kang *et al.* 2013), their effects on plants themselves, particularly on non-crop species, has received much less attention. The results of our study show that antibiotics in concentrations similar to those of agricultural fields had significant effects on the time until germination, on trait-development along ontogenesis and on functional traits of four different plant species.

In our study, absolute rates of germination were similar across all antibiotics and concentrations applied (mean germination rates for *B. napus*: 97.6 %, *C. bursa-pastoris*: 3.25 %, *T. aestivum*: 99.6 % and *A. spica-venti*: 86.6 %, see also Table 3). This lack of an effect on germination is in concordance with most other studies, which used either similar or higher concentrations (Pan and Chu 2016; Ziolkowska *et al.* 2015; Pufal et al. unpublished data; Jin *et al.* 2009; Hillis *et al.* 2011; Yang *et al.* 2010; Liu *et al.* 2009). However, our Kaplan–Meyer survival analysis revealed a significant antibiotic effect on the *time of germination.* Germination rates were generally negatively affected (i.e. delayed, except for the P10 treatment of *B. napus*) when concentration exceeded 1 µg L^−1^, irrespective of the type of antibiotic. This delay was most pronounced for the T10 treatment in *A. spica-venti* (45 h). Thus, it seems that antibiotics in general do not cause lower germination rates *per se*, but trigger a delay in germination. We cannot draw any conclusions on the germination rates of *C. bursa-pastoris*, because this species hardly germinated at all, regardless of treatment. Its very low germination rates may be explained by poor quality seed material or adverse effects of the stratification of 4 °C for 3 days as suggested by Toorop *et al.* (2012), but the precise reasons remain unclear.

In general, delayed seedlings likely face higher competitive pressure, as they need to establish in a community where other plant individuals may already be ahead of them in terms of aboveground and belowground size. This effect may be more severe in natural communities than in cultivated fields. The consequences of delayed germination may become even more pronounced in stressful environments, for example in water-stress environments, which is known from studies on allelopathic effects between plant species, and described as ‘allelopathic retardation’ (Escudero *et al.* 2000 and references therein). We may thus refer to ‘antibiotic induced retardation’ in order to describe a similar pattern induced by antibiotics. However, studies on their effects on community establishment and species composition are still missing.

Germination rates of seeds treated with nitrogen (i.e. N5 and N10) were similar to the control, however, as for antibiotics, there was an effect on the timing of germination. Both grass species showed a significant delay in germination in response to nitrogen addition, with seeds of *T. aestivum* germinating ∼10 h later than those of the control and *A. spica-venti* 21–27 h later, respectively. There was no effect on the two herb species. The study of Pérez-Fernández *et al.* (2006) tested germination rates of eight Mediterranean species to varying levels of pH and nitrogen. Whereas pH did not have an effect on the germination rates, addition of nitrogen (in the forms of NH_4_NO_3_ and KNO_3_, 10 and 50 mM each) decreased the germination rates. Using the same concentration as Pérez-Fernández *et al.* (2006), Rossini Oliva et al. (2009) detected no effect of nitrogen on the germination rates of *Erica andevalensis*, whereas *Lupinus angustifolius* seeds germinated poorer under different types of nitrogen compounds (urea, nitric acid, etc., Kasprowicz-Potocka *et al.* 2013). However, we know of no other study that reports of effects of nitrogen on the timing of germination.

Furthermore, the role of microorganisms on germination and growth of the tested plant species was not taken into account in this study. Soil bacteria are significantly affected by antibiotics (Thiele-Bruhn and Beck 2005; Yang *et al.* 2009), which may in turn affect plant performance and thus plant traits. For example, Yang *et al.* (2010) found an increase in fungi and a decrease in bacteria in response to exposure to tetracycline. Under hydroponic conditions, roots of wheat plants rotted and became atrophic and partly died whereas germination rates were not affected by the treatments. A synchronous inhibition of soil microbial activity and plant biomass production was observed by Wei *et al.* (2009) in a pot trial with tetracycline and ryegrass (*Lolium perenne*). Taking this into account, the results of the present study only reflect to responses of plant traits to the antibiotic treatments, whereas a distinction into direct (uptake and metabolization of the compound by the plant) and indirect (though microbial activity) effects of antibiotics cannot be made.

Our results and the mentioned studies indicate species-specific responses to both antibiotic and nitrogen addition. Both crop species, *B. napus* and *T. aestivum*, germinated most rapidly, followed by the non-crop species *A. spica-venti*, whereas *C. bursa-pastoris* hardly germinated at all. Species-specific responses may be due to differences in seed coats, as pointed out by Pan and Chu (2016) who observed no effect of antibiotics on germination rates, but a linear decrease on root elongation with increasing concentrations of antibiotics. They suggested the seed coat to function as a barrier between the embryo and its environment, which impedes antibiotics from penetrating and affecting the developing individual. However, once germinated, the roots of the young seedling take up antibiotics, which may then subsequently impact growth of the developing plant.

Besides germination, plant traits of later ontogenetic stages were also affected by antibiotics, but effects strongly differed between species as well as between functional plant groups. Significant interactions between species, antibiotics and their concentrations further suggest that the changes in plant traits resulted from species specific responses to the antibiotics. The two herb species both showed clear responses to the treatments, especially in growth and biomass related traits, which were more pronounced for penicillin and sulfadiazine than for tetracycline. In contrast, the two grass species hardly showed any trait responses to antibiotics. The only noteworthy effects were a shift of the Root:Shoot ratio towards a stronger investment in shoot biomass in *T. aestivum*, and negative trait responses in *A. spica-venti*, but only for the highest penicillin treatment. Because previous studies mostly used higher concentrations of antibiotics, we cannot directly compare our results with those studies. Whether grasses are in general less susceptible to natural concentrations of antibiotics than herbs, consequently needs further elucidation.

Post-germinative development of the herb species tested was significantly affected by antibiotics, but effects again differed between the two species and between antibiotics. Canopy height and chlorophyll content (both measured several times in the course of development) responded mostly to penicillin and sulfadiazine, but not to tetracycline. Migliore *et al.* (1997) reported an increase of development-alteration over time, i.e. alterations became more pronounced in later produced plant traits. They tested effects of sulfadimethoxine on root length (lengths of epicotyl, cotyledon and leaves) in *Amaranthus retroflexus*, *Plantago major* and *Rumex acetosella*. In our study, antibiotic effects were more pronounced in later ontogenetic stages for canopy height and more pronounced in earlier stages for chlorophyll content.

**Figure 3:**
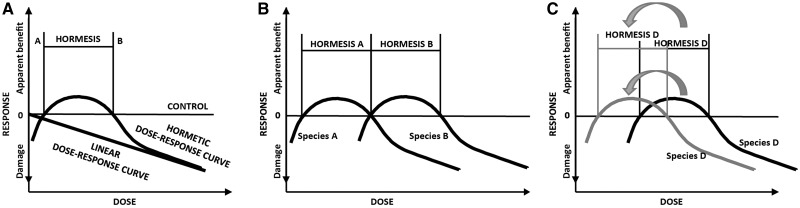
(**A**) Model of non-linear response after Klonowski (2007) and Migliore *et al.* (2010). Damage is caused by deficient doses of an agent (left of A), positive response is caused by low doses (between A and B), while doses exceeding a certain amount cause harmful or toxic effects (right of B). (**B**) Damages and/or hormetic responses (Hormesis A and B) can occur at the same dose-concentrations for different species (Species A and B), respectively. (**C**) Further elaboration of the ‘dilution-effect’ described by Migliore *et al.* (2010). A species that would theoretically show a hormetic response at a certain dose-level shifts its response-interval towards the left side of the concentration gradient, as the agent is diluted by its biomass and tissue water content. The extent of dilution should differ between different species with the same dose-concentration.

Interestingly, effects on chlorophyll content showed different directions for *B. napus* and *C. bursa-pastoris*: whereas pigment content in *B. napus* leaves was lower in treated individuals than in control individuals, it was higher in treated individuals of *C. bursa-pastoris*. The same pattern was found for other functional traits determined at the time of harvest: almost all trait values in *B. napus* were lower than the control, whereas they were higher than the control in *C. bursa-pastoris*. Such opposing patterns have been previously described by two concepts: a) hormesis and b) the dilution effect of biomass (and water) on active substances. Hormesis is a non-linear dose-effect relationship (Klonowski 2007; see Migliore *et al.* 2010 for plant responses towards antibiotics, and Belz and Duke 2014 for resposes towards herbizides) which normally implies that a toxin or pollutant provides a positive stimulus at low doses and inhibition at higher doses (see Fig. 3A for illustration, Calabrese and Baldwin 2002; Calabrese and Blain 2009). Hormesis can occur in all living organisms, including plants (Calabrese and Blain 2009). However, a certain dose that may be beneficial for one individual may be harmful for another or harmful for a population (illustrated in Fig. 3B, Calabrese and Baldwin 2002). With regard to the plant trait responses measured in this study, responses of *B. napus* were mostly negative, whereas those of *C. bursa-pastoris* were mostly positive, indicating species-specific hormetic responses. Whereas the same dose concentration positively stimulated *C. bursa-pastoris* (with trait values lowest for both the lowest and highest antibiotic concentration, compare **[see ****Supporting Information—Table S1****]**), it caused inhibition in *B. napus*.

A ‘dilution-effect’ means that active substances are typically diluted by the aqueous cell components they are dissolved in, which is why they become effective only above a certain species-specific threshold. Consequently, if two species are treated with the same concentration of a certain substance, the species producing higher biomass will also show a higher dilution of the substance, and as a consequence, may differ in its response. Such a ‘dilution-effect’ was observed for e.g. *Lythrum salicaria*-individuals treated with sulfamethoxine (Migliore *et al.* 2010). In their study, individuals of the 0.05 mg L^−1^ treatment showed higher drug tissue concentrations than individuals treated with a concentration of 0.5 mg L^−1^. However, individuals of the 0.5 mg L^−1^ treatment showed higher biomass values and had therefore a lower relative drug concentration, as it was ‘diluted’ by higher biomass and higher water content. In our study, biomass produced by *B. napus* was always higher than that of *C. bursa-pastoris*, except for leaves. Moreover, the response-interval of *B. napus* may have shifted along the dose-gradient to a greater extent than that of *C. bursa-pastoris*, as antibiotics may have been comparatively more diluted (illustrated in Fig. 3C), ultimately leading to the opposing effects between these two species. Testing of the two concepts in a comparative way, however, would require a longer gradient of antibiotic concentrations covering the whole interval of both positive and negative trait responses of all species and a measuring of the antibiotic concentration accumulated in the plant tissue.

## Conclusions

This study demonstrates, as one of the first, that even comparatively small concentrations of antibiotics as typically found in the soil of agricultural landscapes can delay the time of germination and differently affect trait development of different plant species, with effects depending on species, traits, antibiotics and concentrations.

Also, responses were either negative or positive, likely depending on the species, the functional plant group it belongs to or the size (i.e. weight) of an individual (and thus biomass diluting the antibiotics). This relationship between antibiotic-dilution and hormetic responses should be further investigated, as an apparent positive response could result from a diluted toxic effect.

Furthermore, our study revealed that antibiotics in concentrations similar to those detected in grassland soils can have significant effects on the time of germination. If antibiotic effects are indeed largely species-specific, effects of concentrations typically found in real (agricultural) environments could be either negative in some species (i.e. antibiotic induced retardation of germination) or neutral (as in *C. bursa-pastoris*). In this case, less sensitive species may experience a competitive advantage, which might trigger changes in species composition in natural communities. This assumption, however, does not take into account (i) that antibiotics may also accumulate in the soil (Hamscher *et al.* 2002), which can increase total soil concentrations over time and therefore change response patterns in plant communities, (ii) that antibiotics are often found in mixtures in agricultural soils with likely interactive effects between antibiotics and (iii) that antibiotics may also interact with microorganisms in the soil, potentially affecting the response of plants.

This study shows that cropland species can respond to concentrations of antibiotics as typically found in agricultural soils with for example delayed germination or reduced biomass, which may negatively affect yield in farmland fertilized with antibiotic treated manure. Our study also implies that different antibiotics could potentially affect the species composition of natural communities in field margins due to species-specific responses which may affect their competitive abilities. Such species-specific responses may alter the plant species community’s composition, with secondary effects on species of higher trophic levels, like pollinating and herbivorous insects.

## Sources of Funding

This study has been financed by the German Research Foundation (DFG, MI 1365/3-1).

## Contributions by the Authors

V.M., S.L. and G.P. designed the study and raised funding; V.M., A.D. and A.M.V. performed the greenhouse study; V.M. primarily wrote the manuscript with contributions of S.L., G.P., A.D. and A.M.V.

## Conflict of Interest Statement

None declared.

## Supplementary Material

Supplementary Data
