# Supplementary material for: Antibiotics impact plant traits, even at small concentrations
Source: AoB Plants. 2017 Mar 13;9(2):plx010. doi: 10.1093/aobpla/plx010 (PMC5393049; doi:10.1093/aobpla/plx010)
Supplement: Supplementary Data [file plx010_Supp.docx]

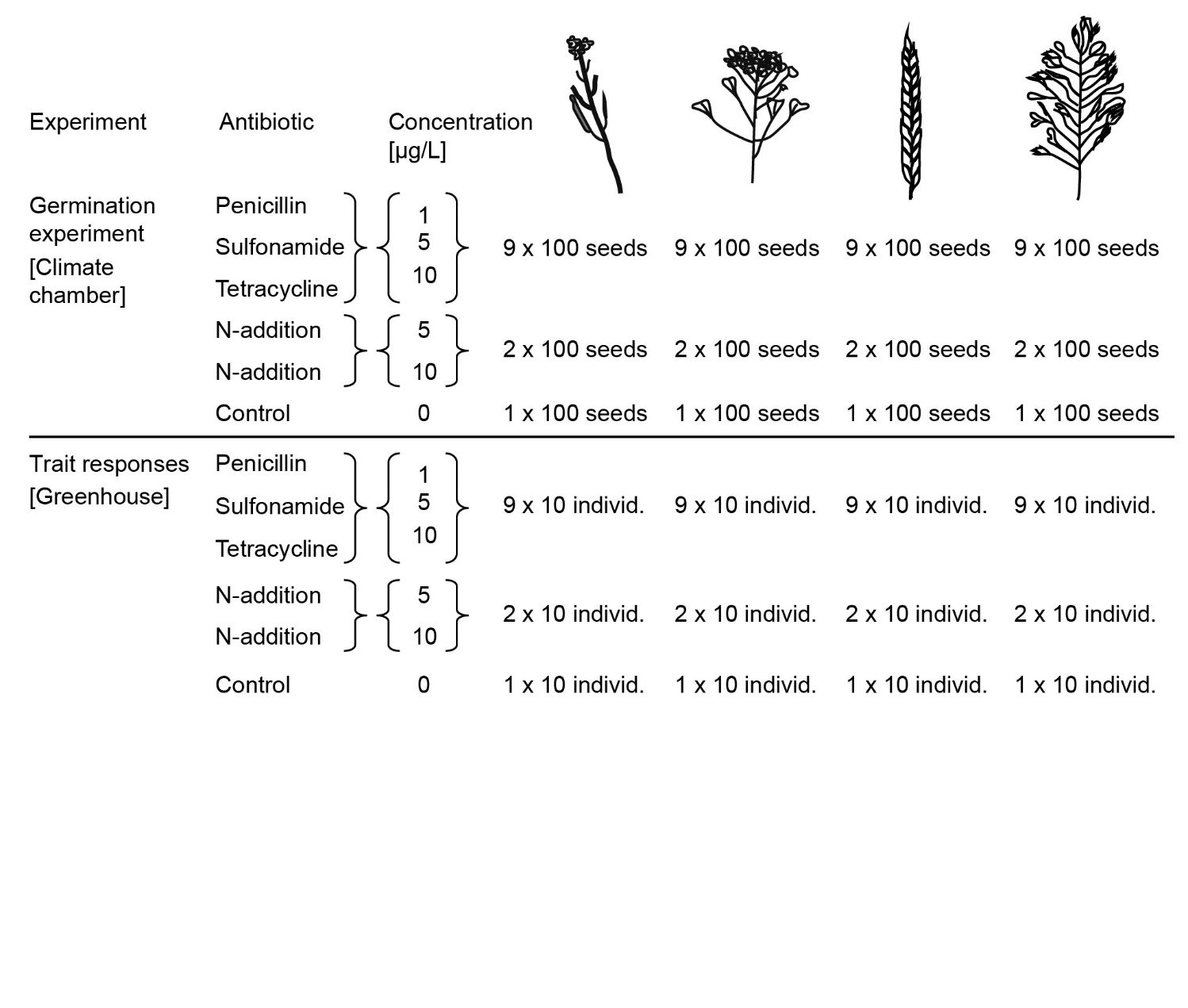


**Figure S1**: Setup of germination experiment (upper part) and greenhouse experiment (lower part). Number of treatments is calculated by the number of antibiotics times the number of concentrations plus control. Plant species are depicted with their inflorescence.

**­Table S1:** Means and relative standard deviations (RSD, %) of each trait for *Brassica napus*, *Capsella bursa-pastoris, Triticum aestivum* and *Apera spica-venti*. Bold numbers indicate significant differences to control treatment (t-test, p<0.05, compare Table 6), green shading indicates significantly lower values, red shading indicates significantly higher values compared to control. Treatments: Control; P1, P5, P10: penicillin treatment in the order 1,5 and 10 µg/L; S1, S5, S10: sulfonamide treatment in the order 1,5 and 10 µg/L; T1, T5, T10: tetracycline treatment in the order 1,5 and 10 µg/L. For abbreviations of traits see Table 2.

| *Brassica napus* | | | | | | | | | | |
| --- | --- | --- | --- | --- | --- | --- | --- | --- | --- | --- |
|  | Control | P1 | P5 | P10 | S1 | S5 | S10 | T1 | T5 | T10 |
| RGR_AGB_ | 0.083 · 4.9 | 0.081 · 5.4 | **0.078 · 5.6** | 0.080 · 4.9 | 0.081 · 6.7 | 0.082 · 4.9 | 0.080 · 3.4 | 0.080 · 8.8 | **0.079 · 8.7** | 0.080 · 4.5 |
| RGR_BGB_ | 0.072 · 8.4 | **0.067 · 11.4** | **0.064 · 6.1** | **0.065 · 10.0** | 0.070 · 10.8 | 0.068 · 7.8 | **0.067 · 6.5** | 0.069 · 13.6 | 0.071 · 12.8 | 0.070 · 7.4 |
| RGR_Total_ | 0.081 · 5.1 | 0.079 · 5.6 | **0.076 · 5.6** | **0.078 · 5.1** | 0.079 · 7.1 | 0.079 · 5.2 | **0.078 · 3.6** | 0.078 · 9.2 | **0.078 · 9.0** | 0.079 · 4.5 |
| Leaf | 684.4 · 21.5 | 599.1 · 24.4 | **492.8 · 24.4** | **536.0 · 19.6** | 597.4 · 25.0 | 597.6 · 23.9 | 579.1 · 20.0 | 634.7 · 26.4 | **582.5 · 34.4** | **556.9 · 16.4** |
| Stem | 662.9 · 32.2 | 561.2 · 34.5 | **490.9 · 26.2** | 573.7 · 39.9 | 580.2 · 39.8 | 654.5 · 25.9 | **498.9 · 17.7** | **519.5 · 43.2** | 524.6 · 35.1 | 558.8 · 29.6 |
| Root | 219.5 · 33.1 | **160.3 · 34.1** | **131.8 · 23.6** | **143.9 · 37.0** | 199.2 · 41.2 | **166.9 · 32.6** | **152.3 · 28.5** | 192.0 · 37.7 | 206.4 · 36.9 | 188.6 · 30.9 |
| StemL | 33.1 · 45.2 | 30.97 · 40.9 | 32.6 · 38.2 | 27.7 · 41.1 | 33.5 · 59.2 | 38.1 · 38.1 | 29.0 · 39.1 | **20.2 · 34.9** | 26.3 · 42.1 | 30.8 · 31.5 |
| SLA | 40.2 · 21.5 | 38.6 · 31.9 | 41.3 · 13.7 | 36.4 · 16.1 | 38.6 · 19.7 | 38.4 · 19.9 | 38.2 · 12.3 | 38.3 · 13.2 | 36.4 · 19.7 | 36.4 · 13.6 |
| Leaf_live_ | 9.5 · 33.3 | **7.2 · 32.6** | 8.2 · 34.4 | **6.3 · 13.1** | 9.3 · 41.8 | 8.7 · 31.2 | 7.8 · 27.6 | **7.0 · 19.1** | 8.5 · 35.6 | 7.7 · 29.4 |
| Leaf_dead_ | 6.0 · 10.7 | 5.9 · 9.6 | 5.7 · 16.6 | 5.9 · 20.3 | 5.8 · 7.3 | 5.9 · 9.6 | 5.9 · 14.8 | 5.9 · 12.5 | 6.1 · 18.0 | 5.7 · 18.6 |
| R:S | 0.16 · 27.3 | 0.14 · 31.8 | 0.14 · 11.3 | **0.13 · 29.3** | 0.17 · 27.6 | **0.13 · 19.4** | 0.14 · 18.8 | 0.17 · 25.6 | 0.19 · 29.1 | 0.17 · 29.9 |
| SRL | 269.8 · 66.9 | **526.2 · 71.1** | 420.4 · 72.4 | 224.7 · 71.1 | 259.9 · 54.1 | 244.7 · 85.3 | 202.8 · 91.8 | 208.1 · 55.8 | 231.9 · 51.0 | 219.3 · 59.3 |
| TRL | 54.9 · 60.4 | 94.3 · 92.2 | 58.9 · 95.2 | **29.7 · 72.8** | 52.7 · 59.5 | 43.6 · 114.6 | **31.9 · 89.9** | 40.8 · 74.7 | 45.9 · 54.4 | 42.2 · 67.2 |
| SecR | 1.41 · 39.5 | **1.91 · 32.1** | **1.90 · 19.0** | 1.39 · 34.4 | 1.38 · 31.5 | **1.82 · 38.3** | 1.67 · 32.9 | 1.32 · 36.1 | 1.41 · 27.0 | 1.47 · 34.5 |
| LPR | 90.4 · 35.9 | 97.0 · 37.5 | 93.7 · 30.0 | **128.3 · 26.3** | 76.8 · 36.7 | 92.3 · 30.2 | 86.6 · 35.3 | 104.6 · 35.0 | 102.4 · 26.6 | 83.1 · 27.8 |
|  |  |  |  |  |  |  |  |  |  |  |
| Capsella bursa-pastoris | | | | | | | | | | |
|  | Control | P1 | P5 | P10 | S1 | S5 | S10 | T1 | T5 | T10 |
| RGR_AGB_ | 0.132 · 3.3 | **0.133 · 3.1** | **0.135 · 1.9** | **0.133 · 4.1** | **0.134 · 5.1** | **0.135 · 3.2** | **0.129 · 4.9** | **0.131 · 2.5** | **0.129 · 4.8** | **0.129 · 6.5** |
| RGR_BGB_ | 0.127 · 6.2 | **0.129 · 4.9** | **0.131 · 4.7** | **0.128 · 8.0** | **0.128 · 8.4** | **0.129 · 6.1** | 0.124 · 9.1 | 0.124 · 4.4 | 0.119 · 9.0 | 0.121 · 10.2 |
| RGR_Total_ | 0.132 · 3.6 | **0.132 · 3.2** | **0.134 · 2.0** | **0.133 · 4.3** | **0.133 · 5.4** | **0.134 · 3.4** | **0.129 · 5.2** | **0.131 · 2.5** | 0.128 · 5.1 | **0.129 · 6.7** |
| Leaf | 688.2 · 42.1 | 661.03 · 28.8 | **831.7 · 23.4** | 707.9 · 32.7 | 699.5 · 35.2 | **768.2 · 29.8** | 662.8 · 44.4 | 663.6 · 23.8 | 538.9 · 35.6 | 587.9 · 42.9 |
| Stem | 260.2 · 39.8 | **298.0 · 18.1** | 241.2 · 63.2 | **312.7 · 30.7** | **353.9 · 27.5** | **311.4 · 23.1** | 190.9 · 57.4 | 232.6 · 52.9 | 268.7 · 46.3 | 281.1 · 34.4 |
| Root | 105.6 · 46.7 | **113.2 · 31.4** | **128.6 · 32.0** | **114.6 · 43.9** | **116.8 · 46.6** | **117.9 · 36.8** | 97.4 · 53.9 | 88.7 · 28.0 | 74.6 · 48.0 | 85.3 · 51.2 |
| StemL | 35.0 · 46.3 | 36.6 · 16.7 | 25.1 · 71.2 | 37.1 · 18.4 | **42.9 · 25.5** | **38.5 · 23.1** | 23.4 · 76.6 | 29.2 · 56.1 | 34.5 · 39.5 | 36.5 · 30.9 |
| SLA | 58.7 · 16.7 | **48.9 · 15.3** | 53.9 · 22.9 | 50.7 · 21.8 | 53.3 · 14.5 | 53.8 · 13.8 | 53.1 · 15.9 | 54.9 · 16.1 | 55.1 · 11.6 | 53.9 · 20.7 |
| Leaf_live_ | 90.7 · 43.9 | **108.6 · 35.7** | 83.5 · 49.9 | 99.8 · 34.3 | **110.0 · 31.8** | 90.6 · 31.2 | 75.1 · 42.4 | 83.9 · 47.4 | **101.4 · 36.6** | **101.9 · 37.4** |
| Leaf_dead_ | 9.3 · 18.9 | **12.2 · 20.0** | 11.5 · 17.9 | **11.6 · 24.5** | 9.8 · 12.5 | 10.7 · 19.3 | 11.2 · 22.2 | 9.0 · 17.6 | 10.6 · 22.3 | 8.7 · 10.9 |
| R:S | 0.11 · 22.8 | 0.12 · 15.5 | 0.12 · 24.4 | 0.11 · 30.8 | 0.11 · 24.3 | 0.11 · 22.8 | 0.11 · 29.5 | 0.09 · 20.1 | 0.09 · 28.3 | 0.09 · 24.9 |
| SRL | 168.9 · 192 | 121.7 · 67.3 | 180.4 · 92.6 | 101.0 · 79.1 | 161.3 · 70.2 | 149.8 · 99.8 | 117.4 · 102 | 139.9 · 90.8 | 117.2 · 57.8 | 190.9 · 123.2 |
| TRL | 16.7 · 188.9 | 13.1 · 68.6 | **20.7 · 77.2** | 11.2 · 98.8 | **21.6 · 90.3** | 18.3 · 112.1 | 10.7 · 126.5 | 14.2 · 110.8 | 9.2 · 89.2 | 12.5 · 114.7 |
| SecR | 1.77 · 36.2 | 1.52 · 37.5 | 1.77 · 39.7 | 1.61 · 41.9 | 1.67 · 37.4 | 1.57 · 47.9 | 1.36 · 30.1 | 1.42 · 28.3 | 1.25 · 25.1 | 1.42 · 25.7 |
| LPR | 150.4 · 29.8 | 147.6 · 21.4 | 165.9 · 16.0 | 122.8 · 25.5 | 134.6 · 14.9 | 160.1 · 33.8 | 140.9 · 43.3 | 143.3 · 23.6 | 158.4 · 28.3 | 138.4 · 31.8 |

*Table S1 - continued*

| *Triticum aestivum* | | | | | | | | | | |
| --- | --- | --- | --- | --- | --- | --- | --- | --- | --- | --- |
|  | Control | P1 | P5 | P10 | S1 | S5 | S10 | T1 | T5 | T10 |
| RGR_AGB_ | 0.042 · 12.3 | 0.041 · 14.4 | 0.043 · 7.0 | 0.043 · 13.3 | 0.045 · 6.6 | 0.041 · 11.6 | 0.042 · 8.2 | 0.039 · 8.9 | 0.041 · 8.2 | 0.043 · 7.1 |
| RGR_BGB_ | 0.014 · 57.6 | 0.018 · 22.4 | **0.019 · 19.6** | 0.019 · 36.7 | **0.019 · 29.4** | 0.015 · 45.5 | 0.018 · 30.7 | **0.009 · 65.9** | 0.015 · 36.0 | 0.017 · 28.4 |
| RGR_Total_ | 0.036 · 15.0 | 0.035 · 15.9 | 0.037 · 8.3 | 0.037 · 16.0 | **0.039 · 8.6** | 0.035 · 14.1 | 0.036 · 10.6 | 0.033 · 10.5 | 0.035 · 9.9 | 0.036 · 9.4 |
| Leaf | 290.9 · 29.2 | 273.3 · 32.9 | 278.9 · 20.1 | 277.5 · 32.2 | 319.4 · 21.1 | 260.6 · 31.5 | 286.0 · 24.5 | 245.9 · 14.9 | 280.6 · 21.4 | 298.6 · 27.3 |
| Stem | 62.6 · 49.8 | 51.2 · 80.1 | 45.9 · 42.5 | 52.5 · 49.7 | 71.5 · 32.7 | 61.6 · 47.0 | 57.6 · 44.8 | 48.1 · 40.1 | 56.3 · 35.2 | 62.6 · 46.4 |
| Root | 47.4 · 48.7 | 52.2 · 26.8 | 54.4 · 25.3 | 55.0 · 37.9 | **60.3 · 35.3** | 47.1 · 45.1 | 55.4 · 31.6 | 35.0 · 22.4 | 46.6 · 26.3 | 51.9 · 32.4 |
| StemL | NA | NA | NA | NA | NA | NA | NA | NA | NA | NA |
| SLA | 34.8 · 13.7 | 36.3 · 13.3 | 34.9 · 19.5 | 38.4 · 8.2 | 34.5 · 7.0 | 34.1 · 16.9 | 35.9 · 15.9 | 37.5 · 22.4 | 36.0 · 6.9 | 35.3 · 16.9 |
| Leaf_live_ | 4.0 · 62.6 | 4.3 · 68.5 | 4.9 · 37.5 | 3.9 · 80.9 | 5.6 · 42.3 | 3.4 · 44.3 | 4.6 · 36.6 | 3.1 · 51.5 | 3.9 · 50.5 | 5.3 · 46.3 |
| Leaf_dead_ | 6.9 · 17.4 | 7.0 · 11.7 | 7.6 · 17.8 | 6.9 · 11.3 | 7.1 · 20.4 | 6.3 · 10.7 | 6.1 · 15.2 | 6.5 · 22.1 | 6.5 · 14.9 | 6.6 · 17.8 |
| R:S | 0.13 · 25.8 | **0.17 · 17.5** | **0.17 · 15.1** | **0.16 · 13.5** | **0.15 · 18.4** | 0.15 · 26.9 | **0.16 · 16.8** | 0.12 · 23.2 | 0.14 · 16.3 | 0.14 · 15.8 |
| SRL | 52.3 · 27.9 | 48.1 · 22.6 | 53.9 · 50.8 | 47.8 · 39.6 | 56.1 · 39.7 | **78.2 · 69.3** | 57.3 · 30.2 | 47.1 · 36.6 | 43.8 · 27.9 | 41.9 · 26.9 |
| TRL | 2.3 · 41.5 | 2.4 · 24.9 | 2.7 · 37.2 | 2.5 · 40.2 | 3.2 · 42.4 | 3.1 · 60.5 | 3.2 · 51.7 | 1.6 · 42.4 | 2.0 · 34.1 | 2.1 · 34.7 |
| SecR | NA | NA | NA | NA | NA | NA | NA | NA | NA | NA |
| LPR | NA | NA | NA | NA | NA | NA | NA | NA | NA | NA |
|  |  |  |  |  |  |  |  |  |  |  |
| Apera spica-venti | | | | | | | | | | |
|  | Control | P1 | P5 | P10 | S1 | S5 | S10 | T1 | T5 | T10 |
| RGR_AGB_ | 0.110 · 10.4 | 0.109 · 11.0 | 0.109 · 7.6 | **0.098 · 16.3** | 0.113 · 5.9 | 0.108 · 7.9 | 0.101 · 17.7 | 0.109 · 18.9 | 0.111 · 6.9 | 0.106 · 12.7 |
| RGR_BGB_ | 0.085 · 22.3 | 0.081 · 18.1 | 0.084 · 13.5 | **0.064 · 45.2** | 0.089 · 11.9 | 0.081 · 16.4 | 0.073 · 35.9 | 0.083 · 45.2 | 0.084 · 17.2 | 0.081 · 30.3 |
| RGR_Total_ | 0.104 · 13.9 | 0.103 · 13.6 | 0.103 · 8.4 | **0.088 · 24.7** | 0.108 · 6.4 | 0.102 · 10.3 | 0.093 · 23.5 | 0.102 · 24.3 | 0.105 · 8.9 | 0.100 · 16.2 |
| Leaf | 199.9 · 35.4 | 156.4 · 44.5 | 188.7 · 47.0 | **122.3 · 66.5** | 190.3 · 30.7 | 172.7 · 39.5 | 142.5 · 66.7 | 213.8 · 60.7 | 189.8 · 32.9 | 166.4 · 51.5 |
| Stem | 64.1 · 46.1 | 47.5 · 55.1 | 58.3 · 44.2 | 45.8 · 60.9 | 70.1 · 41.4 | 51.9 · 50.2 | 46.9 · 52.8 | **86.4 · 50.9** | 66 · 35.3 | 50.6 · 52.4 |
| Root | 40.2 · 46.7 | 25.9 · 43.6 | 39.2 · 68.6 | **22.3 · 69.6** | 36.8 · 44.7 | 30.5 · 56.4 | 28.2 · 74.4 | 62.8 · 76.9 | 38.4 · 50.3 | 37.0 · 65.6 |
| StemL | NA | NA | NA | NA | NA | NA | NA | NA | NA | NA |
| SLA | 57.7 · 11.5 | 62.6 · 16.1 | 56.1 · 12.3 | **51.5 · 14.2** | 60.4 · 10.4 | 56.2 · 15.3 | 54.6 · 11.2 | 57.1 · 7.8 | 54.2 · 13.8 | 60.6 · 18.3 |
| Leaf_live_ | 68.1 · 47.5 | 53.1 · 42.7 | 57.4 · 40.0 | **34.8 · 79.7** | 63.9 · 46.3 | 53.7 · 43.5 | 45.0 · 89.8 | 85.4 · 38.9 | 59.2 · 48.1 | 63.6 · 63.2 |
| Leaf_dead_ | 10.3 · 56.2 | 13.6 · 63.7 | 12.2 · 51.1 | 12.9 · 51.4 | 9.6 · 40.8 | **18.0 · 48.3** | 8.0 · 35.9 | 7.0 · 45.2 | 12.2 · 34.4 | 9.7 · 35.7 |
| R:S | 0.15 · 25.8 | 0.13 · 35.0 | 0.15 · 24.2 | **0.13 · 14.8** | 0.14 · 27.9 | 0.14 · 31.4 | 0.15 · 18.2 | 0.19 · 82.7 | 0.14 · 34.9 | 0.15 · 43.8 |
| SRL | 193.8 · 57.4 | 196.9 · 53.4 | 177.4 · 28.9 | **258.2 · 45.1** | 142.3 · 20.2 | 152.1 · 19.5 | 190.7 · 35.8 | 189.9 · 34.3 | 194.4 · 20.2 | 198.1 · 25.6 |
| TRL | 7.6 · 74.5 | 5.3 · 73.1 | 6.4 · 64.9 | 5.3 · 67.5 | 5.1 · 43.9 | 4.5 · 55.8 | 5.2 · 86.5 | 12.9 · 67.5 | 7.4 · 58.4 | 7.7 · 64.6 |
| SecR | NA | NA | NA | NA | NA | NA | NA | NA | NA | NA |
| LPR | NA | NA | NA | NA | NA | NA | NA | NA | NA | NA |
